# Solila Gold

**Published:** 1896-06

**Authors:** 


### SOLILA GOLD.

Having received numerous inquiries in regard to this gold, we have felt it to be of sufficient interest to have reproduced in this number Dr. Theo. Frick's paper, published in the March number of the *Schweizerische Vierteljahrsschrift für Zahnheilkunde.* Dr. Frick's long connection with that journal,—which we regret to notice has been recently severed,—and his thorough training both in Switzerland and the United States, give his article a special value.

This gold, manufactured by Dr. E. de Trey, of Basel, has slowly been making its way. Our attention was first called to it some two years since. An examination of the sample led at that time to the impression that it probably possessed all the characteristics of crystal or sponge gold, for years familiar to the profession. The interest manifested in it recently, both in England and on the continent, seems to indicate qualities not possessed by older forms. Whether this is true remains to be demonstrated.

